# Comparative Mitogenomic Analysis of Two Snake Eels Reveals Irregular Gene Rearrangement and Phylogenetic Implications of Ophichthidae

**DOI:** 10.3390/ani13030362

**Published:** 2023-01-20

**Authors:** Tianyan Yang, Yuping Liu, Zijun Ning

**Affiliations:** Fishery College, Zhejiang Ocean University, Zhoushan 316022, China

**Keywords:** *Ophichthus*, mitochondrial genome, gene rearrangement, phylogenetic relationship

## Abstract

**Simple Summary:**

It is generally known that the order of mitochondrial genes is highly conserved, but to date, along with the gradual increase of teleostean mtDNA sequences in the GenBank database, the gene rearrangement events have been identified in all the published complete mitogenomes of Ophichthidae species. This amusing finding makes it necessary to carry out studies on mitogenome characteristics and phylogenetic evolution of this fish group. Here, we chose *Ophichthus evermanni* and *O. erabo* as representatives, and obtained the whole mtDNA sequences using the Illumina high-throughput sequencing technology. The novel gene rearrangement and phylogenetic relationships indicated that Ophichthidae might be a monophyletic group and formed a sister group with Muraenesocidae.

**Abstract:**

The family Ophichthidae has the largest number and the most various species (about 359 valid species) in the order Anguilliformes worldwide. Both morphological and molecular characteristics have been used to assess their taxonomic status. However, due to the ambiguous morphological features, molecular data such as mitochondrial DNA sequences have been implemented for the correct identification and classification of these fishes. In this study, the gene arrangement and structure characteristics of two Ophichthidae mitochondrial genomes were investigated for the first time. The total mitogenome lengths of *O. evermanni* and *O. erabo* were 17,759 bp and 17,856 bp, respectively. Comparing with the ancestral mitochondrial gene order, the irregular gene rearrangement happened between *ND6* and tRNA-Pro (P) genes with another similar control region emerging between tRNA-Thr (T) and *ND6* genes, which could be explained by the tandem duplication and random loss (TDRL) model appropriately. ML phylogenetic tree demonstrated that the family Ophichthidae was monophyletic origin, but genus *Ophichthus* might be polyphyletic because of the confused cluster relationships among different species.

## 1. Introduction

The family Ophichthidae comprises 62 genera and 356 species across the globe belonging to two subfamilies, the Myrophinae (70 species) and the Ophichthinae (289 species), respectively. It has the most abundant species of Anguiliformes and represents various morphological characteristics [1]. Ophichthidae fish are widely distributed in the continental shelf of all tropical and subtropical oceans, with the water depths ranging from the intertidal to 1300 m [2]. Their snake-sharped bodies can easily burrow in muddy substrates or coral reefs by hard pointed rayless tail-tips or acute snouts [3]. As the most in quantity and species among eels, the classification and identification of Ophichthidae species is still in confusion because of ambiguous morphological features and variable body shapes at different growth stages [3,4]. Since the concept of molecular systematics was first proposed in 1965, it has been considered a powerful tool to solve the traditional taxonomic problems and provide new insights in phylogenetic relationships of living organisms [5]. Mitochondrial DNA (mtDNA) is one of the most valuable and popular molecular markers in the fields of molecular systematics, population genetics and evolutionary biology at multiple taxonomic levels [6,7].

The typical mitochondrial genome is a covalently double-stranded cyclic DNA and normally consists of two ribosomal RNAs (rRNAs), 22 transfer RNAs (tRNAs), 13 protein-coding genes (PCGs), and non-genic regions (control region and the origin of light strand replication). The sequential order of genes in mitochondrial DNA initially tends to be conserved in metazoan [8]. However, in recent years, cases of mitochondrial rearrangement have subsequently been reported in some amphibians [9,10], reptiles [11,12,13], aves [14,15,16], and arthropods [17,18], with the rapid development of sequencing technology. Intraspecific rearrangements of mitogenomes are rarely found in bony fishes, but certain flatfishes [19,20,21,22,23], Antarctic notothenioids [24,25], and deep-sea fishes [26,27] had the rearranged gene order. According to the teleostean complete mtDNA sequences published in NCBI database, the probabilities of gene rearrangements are much higher in Anguiliformes. The previous research has been performed in the systematic evolution of Elopomorpha fishes based on mtDNA genes [28,29,30,31,32]. Nevertheless, individual gene markers of partial fragments may not supply adequate evidence to explain the kinships among Anguiliformes, especially phylogenetic relationships of the teleost fishes grouped into the family Ophichthidae have been poorly understood. 

Variability of mitochondrial gene order among animal phyla contains quantitatively meaningful information for phylogenetic reconstruction. Therefore, gene arrangement is considered a useful phylogenetic character [33,34]. In this study, we obtained the complete mitochondrial DNA sequences of two representative snake eels *Ophichthus evermanni* (Jordan & Richardson, 1909) and *O. erabo* (Jordan & Snyder, 1901) using next-generation sequencing (NGS) technology for the first time, and a novel mitochondrial reorganization was discovered in comparison with normal gene order in other vertebrate lineages. Simultaneously, 23 rearranged complete mtDNA sequences of Anguilliformes available from GenBank were downloaded to construct the phylogenetic tree. The comparative mitogenomics results would provide references for further molecular identification and phylogenetics research on Ophichthidae.

## 2. Materials and Methods

### 2.1. Sampling, DNA Extraction, and High-Throughput Sequencing

Specimens of *O. evermanni* (female, body length 77.1 cm and body weight 571.63 g) and *O. erabo* (male, body length 73.44 cm and body weight 389.32 g) were captured from the coastal waters of Xiamen (118°34′ E, 24°15′ N) China in December 2020 and January 2021, respectively. The snake eels were characterized and identified using both morphometric and molecular methods after euthanizing them with 200 mg/L tricaine methanesulfonate (MS-222) solution for about 20 min [35]. Fresh muscle tissues were clipped from the body and immediately preserved in absolute ethanol for long-term storage. 

The genomic DNA was extracted according to standard phenol-chloroform method [36], and then assessed by 1% agarose gel electrophoresis and Qubit 2.0 (Invitrogen, CA, USA), respectively. The quantified DNA samples were randomly broken into small fragments (about 300 bp) using Covaris M220 Focused-ultrasonicator (Covaris, MA, USA). The paired-end libraries were constructed and subsequently sequenced via 300 bp paired-end Illumina HiSeq 2500 platform following manufacturer’s recommendations.

### 2.2. Mitogenome Assembly and Annotation

The raw data were filtered by clipping adapters, duplicated reads and short reads (<50 bp), the reads with proportion of uncertain bases “N” exceeding 10% and low quality reads (<Q20) using Trimmomatic (http://www.usadellab.org/cms/index.php?page=trimmomatic (accessed on 15 March 2022)) [37]. Multiple iterations and splicing were performed to assemble the mitochondrial genome by Getorganells (https://github.com/Kinggerm/GetOrganelle (accessed on 3 April 2022)) [38]. Bases correcting, mis-assemblies fixing, and gaps filling were conducted by Pilon 1.23 [39]. The complete mitochondrial genome sequences were annotated with the online tool Mitofish (http://mitofish.aori.u-tokyo.ac.jp/ (accessed on April 18 2022)) and the annotation results were checked manually for the further analysis. The entire mitogenome sequences were deposited to GenBank database using Sequin, under the accession numbers OM421636 for *O. evermanni* and OP154196 for *O. erabo*.

### 2.3. Sequence Analysis

Protein-coding genes (PCGs) were examined by translating into their corresponding amino acid sequences to correct premature or truncated stop codons using MEGA 11 [40]. The tRNA genes were predicted and identified using the default search mode of the online tool tRNAscan-SE (http://lowelab.ucsc.edu/tRNAscan-SE/ (accessed on 4 May 2022)) [41] and their cloverleaf structures were visualized by diagram online tool tRNAdb (http://rna.tbi.univie.ac.at/forna/ (accessed on 8 May 2022)) [42]. The location of origin of light-strand replication (O_L_) was identified via sequence homology alignment, and the putative secondary structure was inferred using Mfold web server (http://www.unafold.org/ (accessed on 10 May 2022)) [43]. The pattern graphs for gene order rearrangement were designed and drew by IBS 1.0 [44].

The nucleotide and amino acid composition calculations and the relative synonymous codon usage (RSCU) analysis were performed in MEGA 11 [40], and nucleotide composition skewness was calculated by the following formulas: AT-skew = (A − T)/(A + T) and GC-skew = (G − C)/(G + C) [45]. The values of nonsynonymous substitution (*Ka*)/synonymous substitution (*Ks*) were determined by KaKs_Calculator 2.0 software (https://ngdc.cncb.ac.cn/biocode/tools/BT000001 (accessed on 2 June 2022)) [46].

### 2.4. Phylogenetic Analysis

Twenty-three Anguilliformes mitogenomes existing gene rearrangements were downloaded from the GenBank database for phylogenetic analysis, with *Neocyema erythrosoma* (AP018345) belonging to Saccopharyngiformes, Cyematidae be selected as an outgroup. Twelve PCGs without stop codons were concatenated for the phylogenetic analysis. The *ND6* gene was excluded because of its poor phylogenetic performance [47,48]. Multiple sequences were aligned by MegAlign program in DNAstar Lasergene software package [49]. The use of the maximum likelihood (ML) method in developing phylogenetic requires a model of evolution [50]. Therefore, a general-time reversible + gamma + invariants (GTR + G + I) nucleotide substitution model was selected from the Model Test conducted using MEGA before phylogenetic inference. DAMBE 5.0 was taken to measure the nucleotide substitution saturation [51], and then PhyML 3.0 was used to construct the phylogenetic tree with node support being calculated by 1000 bootstrap replicates to estimate confidence of tree topology [52]. The graphic representation was performed and manually edited with iTOL (https://itol.embl.de/ (accessed on 18 June 2022)).

## 3. Results

### 3.1. Mitogenome Characteristics and Organization

**Figure 1:**
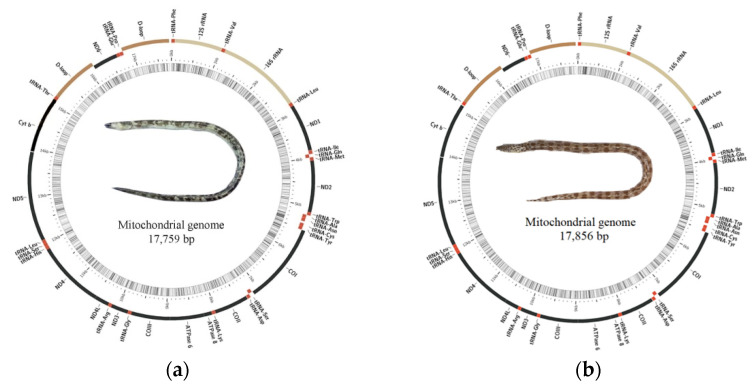
Circular mitochondrial genome maps of two *Ophichthus* fishes. (**a**) *O. evermanni* and (**b**) *O. erabo*.

The total lengths of the mitochondrial genomes were 17,759 bp (*O. evermanni*) and 17,856 bp (*O. erabo*), respectively (Figure 1 and Table 1). Only *ND6* and eight tRNA genes were encoded on the L-strand. A total of 78 bp and 98 bp intergenic gaps were observed, ranging from 1 to 18 bp. The largest overlap detected between *ATP8* and *ATP6* genes was 10 bp in length, followed with 7 bp overlap between *ND4L* and *ND4* genes. The similarity of entire PCGs between two species varied from 76.5% (Cyt *b*) to 87.0% (*COII*).

The proportions of four nucleotides were listed as follows: A = 31.27%, G = 16.19%, C = 26.22%, T = 26.32% in *O. evermanni*, and A = 32.07%, G = 16.25%, C = 26.24%, T = 25.44% in *O. erabo*, respectively, showing approximate equilibria between C and T as well as an obvious bias of A. The contents of A and C exhibited high values at the third codon position, indicating that the codon usage preferred A and C at this position. All the AT-skews were positive except six genes (CRs, *COI*, *ATP6*, *COIII*, *ND3,* and *ND6*) in *O. evermanni,* and five genes (*ND1*, *COI*, *ND3*, *ND4L,* and Cyt *b*) in *O. erabo*, whereas negative GC-skews were lowest in ATP8 and highest in tRNAs both in two snake eels (Table 2).

The nucleotide composition and skewness of 25 Anguilliformes mitogenome were analyzed in Supplementary Table S1. The results showed those overall base compositions of two fishes possessed a higher A + T bias of 57.59% (*O. evermanni*) and 57.50% (*O. erabo*), indicating positive AT-skew and negative GC-skew. The average AT-skews of twenty-five eels was 0.108, ranging from 0.069 in *Muraenesox bagio* to 0.170 in *Paraconger notialis*, while the average GC-skew was −0.230, ranging from −0.261 in *Thalassenchelys* sp. to −0.164 in *Conger japonicas*. 

### 3.2. Novel Gene Arrangement and the Possible Pathway

The arrangement of mtDNA genes of two fishes deviated from the classic vertebrate type. *ND6* and tRNA-Glu genes were located between tRNA-Thr and tRNA-Pro genes, and another highly homologous control region appeared in the upstream of the *ND6* gene. Excluding *ND6*, the tRNA-Glu translocation and CR repeat sequence of the remaining genes was consistent with normal mitochondrial genomic order in the vertebrate (Figure S1). The hypothesized intermediate steps of gene rearrangement were as follows. First, the gene cluster “*ND6*–tRNA-Glu (E)–Cyt *b*–tRNA-Thr (T)–tRNA-Pro (P)–CR” was tandemly duplicated, and then random deletions of redundant genes containing original *ND6*, tRNA-Glu (E), tRNA-Pro (P) as well as the copied Cyt *b* and tRNA-Thr (T) occurred, which most likely resulted in the observed gene order and associated intergenic spacers in *O. evermanni* and *O. erabo*. 

### 3.3. Protein Coding Genes (PCGs) and Codon Usages

The total lengths of the PCGs in *O. evermanni* and *O. erabo* were 11,519 bp and 11,518 bp, respectively, accounting for 64.86% and 64.50% of the whole sequences. The A + T contents in the regions were 57.40% and 56.90%, which were slightly lower compared with that of the entire mitogenomes. Lower guanine content and higher adenine content were exhibited in the 1st codon position, while the 2nd position did not display deflection of A, T and C proportions. An anti g bias was noted in the 3rd codon position, which was as same as the typical vertebrates [53].

Just like other bony fishes, the 12 PCGs in the mtDNA of two snake eels were located on the majority strand excluding the *ND6* gene. The start codons were completely consistent with each other, and all of them started with the typical ATG, except for the codon for the *COI* gene, which used the GTG and *ND6* gene with CTA as the initiation codon. In both species *ND2*, *ND3*, *ND4,* and *COII* ended with the incomplete stop codon T--, yet in *O. evermanni*, *ATP6* and Cyt *b* used TA- and AA- as termination codons, respectively, which would be completed by adding a poly A tail during RNA processing and act as the complete functional termination codons in the processes of polyadenylation and polycistronic transcription cleavage [54]. 

**Figure 2:**
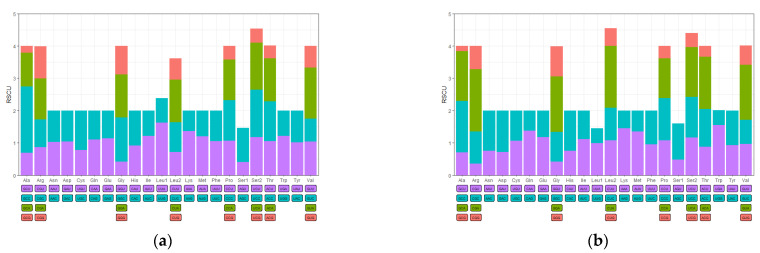
Relative synonymous codon usages (RSCUs) in the mitogenomes of (**a**) *O. evermanni* and (**b**) *O. erabo.* Bars of different colors correspond to the codons with the same color.

A total of 3658 (*O. evermanni*) and 3657 (*O. erabo*) amino acids were present in the 13 PCGs without the stop codons, respectively. Two fish species owned the similar codon usage with the most commonly used amino acids being Leu (13.48% in *O. evermanni* and 13.50% in *O. erabo*), while the Cys (1.46% in *O. evermanni* and 1.73% in *O. erabo*) was the most rarely used (Figure S2). The content of AT-rich codon families (Phe, Ile, Met, Tyr, Asn, and Lys) was higher than that of GC-rich codon families (Pro, Ala, Arg, and Gly). The relative synonymous codon usage (RSCU) values of PCGs were summarized in Figure 2. All stop codons were excluded from the analysis to avoid biases due to incomplete stop codons. The frequency of use of codons has no difference from other degenerate codons if the RSCU value equals 1 [55], and it was found that most of the over-represented codons were A and U ending types (RSCU > 1) of PCGs in the mitogenomes of *O. evermanni* and *O. erabo*.

### 3.4. Ribosomal and Transfer RNA Genes

Next, 12S rRNA and 16S rRNA genes were situated between tRNA-Phe (F) and tRNA-Leu^UUA^ (L1) with tRNA-Val (V) sandwiched in between them, which was similar to other vertebrate mitogenomes to date [47,53]. The lengths of rRNAs occupied a sum of 14.99% (2662 bp) and 14.96% (2672 bp) of the whole mitochondrial genomes, with positive AT-skew values as 0.279 and 0.303, respectively. The A + T content of 16S rRNA gene (57.25% in *O. evermanni* and 58.42% in *O. erabo*) was slightly greater than that of 12S rRNA gene (53.39% in *O. evermanni* and 53.26% in *O. erabo*), both contributing to the overall total rRNAs A + T content of 55.86% and 56.55%.

The size range of 22 tRNAs was from 65 bp for tRNA-Cys (C) to 76 bp for tRNA-Leu^UUA^ (L). Majority tRNAs placed in H-strand while minority of them were in L-strand. Two kinds of tRNA-Leu (L) and tRNA-Ser (S) were discovered in the mtDNA of both species with the anticodons listed: tRNA-Leu^UUA^ (L1) = TAA, tRNA-Leu^CUA^ (L2) = TAG, tRNA-Ser^UCA^ (S1) = TGA, and tRNA-Ser^AGC^ (S2) = GCT. The comprehensive secondary structures of tRNA genes for both *O. evermanni* and *O. erabo* were shown in Supplementary Figure S3. The traditional clover-leaf structures of tRNA genes were identified, including amino acid receptor arm, anticodon arm, pseudouracil (TΨC) arm and dihydrouracil (DHU) arm, and their connected loops. Only tRNA-Ser^AGC^ (S2) was an exception because it was lacking the DHU arm. In addition to standard Watson–Crick base pairs (A-U and G-C), there were 36 bp mismatches present in the tRNA secondary structures, all of which formed a noncanonical bond (G-U pairs). Moreover, DHU and TΨC arms were seemed to be more conserved than the other two in consideration of the lower variation of base pairs.

### 3.5. Noncoding Regions

In this study, the noncoding regions included the light strand origin (O_L_) as well as two highly homologous control regions (CRs), and this phenomenon was noticed in the published mitochondrial genome of *O. brevicaudatus* [56]. The ancestral CR was still located between tRNA-Pro (P) and tRNA-Phe (F), as well as the duplicated one was between tRNA-Thr (T) and *ND6* gene. The O_L_ is situated within a tRNA gene cluster, which is often referred to as the WANCY region [57]. It was 21–34 bp to long in nine Ophichthidae species, and the single stranded DNA folding pattern showed that this region possibly formed a stable hairpin structure with a GC rich stem and a 5–12 bp small loop in length (Table S2). The use of stem codons showed more pyrimidines at the 5′ end of the O_L_ sequence. No typical conserved sequence motif 5′-GCCGG-3′ was detected in the upstream of the stem, which was necessary for vitro replication of the L-strand in mammals [58,59].

### 3.6. Selective Pressure Analysis of PCGs

The nonsynonymous substitution (*Ka*) and synonymous substitution (*Ks*) of protein-coding sequences are two important parameters to quantify the selective pressure and evolutionary dynamics across closely related species [60]. The ratios of *Ka*/*Ks* of two snake eels were calculated to determine selective pressure levels imposed on 13 PCGs. We found the ratios of the 12 PCGs were all below 1, ranging from 0.0113 (*COI*) to 0.3302 (Cyt *b*), indicating a strong repair mechanism against deleterious mutations on these protein genes. Only *ND6* gene presented the value larger than 1 (Figure S4), which demonstrated that it was subject to positive selection. 

### 3.7. Phylogenetic Analysis

ML phylogenetic tree were constructed on the basis of linked sequences of 12 PCGs (without stop codons). DAMBE was conducted to test the nucleotide substitution saturation prior to phylogenetic analysis. The observed *I*_ss_ value was 0.4760, and it was smaller than *I*_ss.c_ values both in assuming a symmetrical tree (0.8495) and assuming an extreme asymmetrical tree (0.6262) when all three codon positions were considered as a whole. 

**Figure 3:**
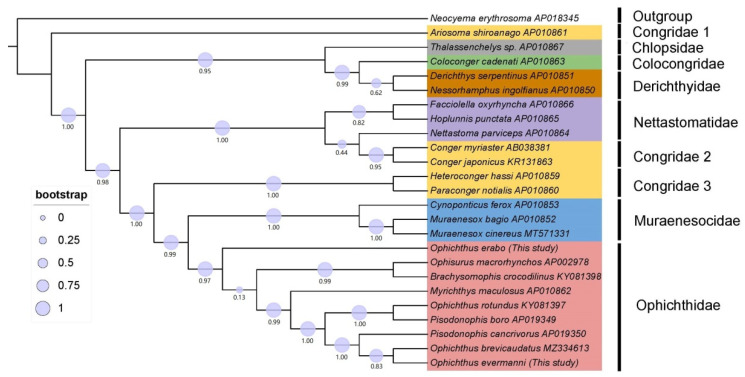
Phylogenetic tree of 25 Anguilliformes species constructed by Maximum Likelihood (ML) method based on concatenated sequences of 12 PCGs.

The reconstructed ML tree revealed that all Ophichthidae species gathered together, but the clustering relations among genera within this family were confused (Figure 3). According to the taxonomic classification, species belonging to the genus of *Ophichthus* were supposed to cluster in the same topological clade, whereas *O. erabo* was even outermost-situated at the clade of Ophichthidae. The family Ophichthidae was monophyletic and formed a sister group with Muraenesocidae under a higher bootstrap support rate (97%). 

## 4. Discussion

Three indexes of A + T content, AT-skew, and GC-skew were usually used to describe the patterns of nucleotide composition and strand asymmetry in DNA sequences, especially when the latter two could clearly reflect the strand bias in nucleotide composition of metazoan mitogenomes [61]. In the present study, the GC-skew values of the 13 PCGs among the two species were negative, demonstrating base C were more plentiful than G. Moreover, the absolute values of GC-skew were larger than those of AT-skew, conforming to conventional preferences that GC-skew was more obvious [62]. A strong AT-bias in the 3rd codon position and the preferred codons were influenced by compositional constraints. The results confirmed that codon usage bias and the AT bias of the 3rd codon position were positively correlated in the mitogenomes [63,64].

Shuffling, translocation, and inversion are the main types of gene reorganization of fish mitogenomes [65]. Six models have hitherto been proposed to explain the rearrangement mechanism: tandem duplication and random loss (TDRL), tandem duplication and nonrandom loss (TDNL), dimer-mitogenome and nonrandom loss (DMNL), double replication and random loss (DRRL), intra-mitochondrial recombination and tRNA mis-priming [20,21]. Among the available models for gene rearrangement events, the TDRL model that was assumed to be triggered by gene duplication was the most appropriate one to explicate the gene rearrangement of Anguilliformes mitogenomes [56,66,67,68]. According to this mode, it was speculated that the tandem duplication and subsequent deletion events occurred between the *ND6* gene and CR both in *O. evermanni* and *O. erabo*.

Noncoding regions are scattered through the whole genome in spite of compact mitogenome of typical teleosts [69]. Variations (duplication or degeneration) in CR, the large non-coding region (LNR) in mtDNA have very important evolutionary values in metazoan mitochondria, because it contains regulatory elements involving in mtDNA replication and transcription [70]. Two separate CRs with identical or highly similar nucleotide sequences are rare in vertebrates. Duplicate CRs have been happened in the mitogenomes of some birds [71,72,73], turtles [74], snakes [11,75], and frogs [76] to date. In ray-finned fishes, Lee et al. [77] described two CRs in mtDNA of *Rivulus marmoratus* for the first time, and subsequently a deep-sea gulper eel *Saccopharynx lavenbergi* [27], three flatfishes (*Crossorhombus azureus, Samariscus latus* and *Laeops lanceolata*) [19,78], and a newly named pomfret *Pampus minor* [79] displayed the similar mitogenomic structure. In our study, two CRs with higher similarity (94.5% in *O. evermanni* and 88.6% in *O. erabo*) were found in two *Ophichthus* species. At present, hypotheses such as tandem duplication, dimerization, and illegitimate recombination are proposed to explain how duplicated CRs are generated [80]. Additionally, it is reported that duplicate CRs have evolved in either concerted evolution [11,70,75,78], independent evolution [72], or both [70,71] in fishes and other animals. The possible evolutionary pattern and originated mechanism of the duplicate CRs still need more specimens for deeper analysis.

For most proteins, *Ka = Ks* means neutral mutation, *Ka < Ks* indicates negative (purifying) selection and *Ka > Ks* suggests a positive (diversifying) selection, respectively [81,82]. It was revealed that only *ND6* gene experienced positive selection in our study. The proton-translocating NADH-quinone oxidoreductase (Complex I), a multisubunit integral membrane enzyme, catalyzes electron transfer from NADH through the respiratory chain of both bacteria and eukaryotic organelles. In addition, it provides about 40% of the proton-motive force required to synthesize ATP in vertebrates [83,84]. Mitochondrial DNA-encoded *ND6* subunit is an indispensable part of Complex I. As the benthic and cave-dwelling fishes with a preference for slithering in muddy substrates, mutations leading to amino acid changes of *ND6* imply that they are undergoing evolutionary selection associated with hypoxia tolerance in snake eels [48,85]. Besides, larger fluctuation in AT/GC-skew value in *ND6* also suggested that the mutational pressure on it might be significantly different from other genes from another perspective.

In this study, the test of substitution saturation indicated the set of aligned nucleotide sequences experienced little substitution saturation and consequently phylogenetic analysis was feasible [86]. These findings of molecular phylogenetic analysis coincided with previous taxonomic studies on snake eels [87,88], which showed that Ophichthidae fishes occurring mitochondrial gene rearrangements should be diagnosed as a monophyletic group because of the highly unlikely homoplasy [33], and evidence supplied by other researchers also validated this conclusion [56,66,68].

## 5. Conclusions

The current study provided the mitogenomic characteristics of two snake eels (*O. evermanni* and *O. erabo*) from Xiamen offshore sea by high-throughput sequencing technology, with the total lengths of 17,759 bp and 17,856 bp, respectively. Different from the typical bony fishes, the complete mtDNAs of both species contained 37 genes (2 rRNAs, 22 tRNAs and 13 PCGs), two CRs, and the O_L_. Moreover, a novel gene arrangement obviously occurred in the region from *ND6* to CR, and the presumptive arrangement mechanism could be explained by TDRL model. The present gene order and associated intergenic spacers could have resulted from an occurrence of duplication in this gene cluster followed by random deletion of five redundant genes *ND6*, Cyt *b*, tRNA-Glu (E), tRNA-Thr (T) and tRNA-Pro (P). The ratio of *Ka* and *Ks* indicated that only *ND6* gene underwent a diversifying selection, which might be related to adaptive evolution of hypoxia tolerance in this fish group. Phylogenetic analysis through 25 Anguilliformes mitochondrial genomes with gene rearrangements showed that Ophichthidae was monophyletic origin and had close relationships with Muraenesocidae. In summary, our research results provided useful information for in-depth understanding of phylogenetics and evolutionary mechanism in family Ophichthidae. 

## Figures and Tables

**Table 1:** Features of the mitochondrial genomes of two *Ophichthus* species.

Gene	Direction	Strand	Start to End	Size/bp	Intergenic Length/bp	Start Codon	Stop Codon	Anticodon	Similarity/%
			*O. evermanni/O. erabo*	*O. evermanni/O. erabo*	*O. evermanni/O. erabo*	*O. evermanni/O. erabo*	*O. evermanni/O. erabo*	*O. evermanni/O. erabo*	
tRNA-Phe (F)	+	H	1–68/1–69	68/69				GAA/GAA	85.5
12S rRNA	+	H	69–1027/70–1036	959/967					86.5
tRNA-Val (V)	+	H	1028–1098/1037–1107	71/71				TAC/TAC	94.4
16S rRNA	+	H	1099–2801/1108–2812	1703/1705					84.6
tRNA-Leu^UUA^ (L1)	+	H	2802–2877/2813–2888	76/76				TAA/TAA	90.8
*ND1*	+	H	2878–3846/2889–3857	969/969		ATG/ATG	TAA/TAG		79.3
tRNA-Ile (I)	+	H	3850–3922/3861–3933	73/73	3/3			GAT/GAT	89.0
tRNA-Gln (Q)	-	L	3922–3992/3933–4003	71/71	−1/−1			TTG/TTG	90.1
tRNA-Met (M)	+	H	3992–4060/4003–4071	69/69	−1/−1			CAT/CAT	85.5
*ND2*	+	H	4061–5117/4072–5128	1057/1057		ATG/ATG	T--/T--		76.7
tRNA-Trp (W)	+	H	5118–5186/5129–5200	69/72				TCA/TCA	86.1
tRNA-Ala (A)	-	L	5188–5256/5202–5270	69/69	1/1			TGC/TGC	85.5
tRNA-Asn (N)	-	L	5258–5330/5272–5344	73/73	1/1			GTT/GTT	94.5
O_L_	+	H	5335–5368/5351–5376	34/26	4/6				61.8
tRNA-Cys (C)	-	L	5375–5439/5394–5459	65/66	6/17			GCA/GCA	86.4
tRNA-Tyr (Y)	-	L	5440–5510/5460–5530	71/71				GTA/GTA	85.9
*CO I*	+	H	5512–7152/5532–7172	1641/1641	1/1	GTG/GTG	TAG/TAA		84.5
tRNA-Ser^UCA^ (S1)	-	L	7169–7239/7191–7261	71/71	16/18			TGA/TGA	95.8
tRNA-Asp (D)	+	H	7245–7312/7267–7334	68/68	5/5			GTC/GTC	88.2
*CO II*	+	H	7319–8009/7341–8031	691/691	6/6	ATG/ATG	T--/T--		87.0
tRNA-Lys (K)	+	H	8010–8084/8032–8106	75/75				TTT/TTT	90.7
*ATP8*	+	H	8086–8253/8108–8275	168/168	1/1	ATG/ATG	TAA/TAA		80.5
*ATP6*	+	H	8244–8926/8266–8949	683/684	−10/−10	ATG/ATG	TA-/TAA		78.9
*CO III*	+	H	8927–9712/8949–9734	786/786	0/−1	ATG/ATG	TAA/TAA		85.2
tRNA-Gly (G)	+	H	9712–9783/9734–9805	72/72	−1/−1			TCC/TCC	86.1
*ND3*	+	H	9784–10132/9806–10154	349/349		ATG/GTG	T--/T--		81.1
tRNA-Arg (R)	+	H	10133–10202/10155–10224	70/70				TCG/TCG	97.1
*ND4L*	+	H	10203–10499/10225–10521	297/297		ATG/ATG	TAA/TAA		85.5
*ND4*	+	H	10493–11873/10515–11895	1381/1381	−7/−7	ATG/ATG	T--/T--		80.7
tRNA-His (H)	+	H	11874–11942/11896–11964	69/69				GTG/GTG	95.7
tRNA-Ser^AGC^ (S2)	+	H	11943–12012/11965–12034	70/70				GCT/GCT	67.5
tRNA-Leu^CUA^ (L2)	+	H	12013–12085/12035–12107	73/73				TAG/TAG	95.9
*ND5*	+	H	12086–13921/12108–13943	1836/1836		ATG/ATG	TAA/TAA		79.3
Cyt *b*	+	H	13936–15077/13959–15098	1142/1140	14/15	ATG/ATG	AA-/TAA		76.5
tRNA-Thr (T)	+	H	15095–15166/15116–15187	72/72	17/17			TGT/TGT	91.7
CR1	+	H	15167–16132/15188–16224	966/1037					67.1
*ND6*	-	L	16133–16651/16225–16743	519/519		CTA/CTA	CAT/CAT		77.3
tRNA-Glu (E)	-	L	16652–16720/16744–16812	69/69				TTC/TTC	85.5
tRNA-Pro (P)	-	L	16724–16794/16820–16890	71/71	3/7			TGG/TGG	85.9
CR2	+	H	16795–17759/16891–17856	965/966					72.4

**Table 2:** Composition and skewness of mitogenomes in two *Ophichthus* species.

	*O. evermanni/O. erabo*						
	A%	T%	G%	C%	A + T%	AT-Skew	GC-Skew
tRNAs	30.93/31.60	24.63/24.81	20.13/19.68	24.31/23.91	55.56/56.41	0.113/0.120	−0.094/−0.097
rRNAs	35.73/36.83	20.14/19.72	19.68/19.57	24.46/23.88	55.86/56.55	0.279/0.303	−0.108/−0.099
CRs	31.75/36.45	31.85/27.36	15.69/15.08	20.71/21.12	63.59/63.80	−0.002/0.142	−0.138/−0.167
*ND1*	27.04/26.21	26.42/26.73	15.79/17.54	30.75/29.51	53.46/52.94	0.012/−0.010	−0.321/−0.254
*ND2*	34.56/34.34	24.27/24.60	12.65/13.25	28.52/27.81	58.83/58.94	0.175/0.165	−0.385/−0.355
*COI*	27.54/27.48	28.34/28.40	18.22/18.46	25.90/25.66	55.88/55.88	−0.014/−0.016	−0.174/−0.163
*COII*	29.23/31.11	28.80/27.06	16.79/15.48	25.18/26.34	58.03/58.18	0.007/0.070	−0.200/−0.260
*ATP8*	35.12/36.31	25.60/27.38	11.31/8.33	27.98/27.98	60.71/63.69	0.157/0.140	−0.424/−0.541
*ATP6*	29.28/28.95	30.60/27.63	12.15/12.57	27.96/30.85	59.88/56.58	−0.022/0.023	−0.394/−0.421
*COIII*	27.35/27.35	27.48/26.59	17.56/18.45	27.61/27.61	54.83/53.94	−0.002/0.014	−0.222/−0.199
*ND3*	28.37/24.36	30.37/32.95	13.47/16.05	27.79/26.65	58.74/57.31	−0.034/−0.150	−0.347/−0.248
*ND4L*	28.62/27.61	27.27/27.95	13.80/13.80	30.30/30.64	55.89/55.56	0.024/−0.006	−0.374/−0.379
*ND4*	30.27/30.99	27.52/26.72	14.12/13.61	28.10/28.67	57.78/57.71	0.048/0.074	−0.331/−0.356
*ND5*	31.81/33.33	26.96/26.25	13.56/13.34	27.67/27.07	58.77/59.59	0.083/0.119	−0.342/−0.340
Cyt *b*	39.50/28.07	14.26/28.68	14.07/16.40	32.18/26.84	53.76/56.75	0.469/−0.011	−0.392/−0.241
*ND6*	29.42/40.66	30.56/12.91	14.80/13.49	25.22/32.95	59.98/53.56	−0.019/0.518	−0.260/−0.419
PCGs	30.23/30.34	27.17/26.56	14.89/15.21	27.71/27.89	57.40/56.90	0.053/0.066	−0.301/−0.294
1st codon site	29.61/30.34	28.43/24.71	14.89/18.07	27.08/26.88	58.04/55.05	0.020/0.102	−0.290/−0.196
2nd codon site	30.44/26.84	28.46/31.72	13.07/14.51	28.02/26.94	58.90/58.56	0.034/−0.083	−0.364/−0.299
3rd codon site	30.64/33.90	24.62/23.21	16.70/13.01	28.04/29.87	55.26/57.11	0.109/0.187	−0.253/−0.393

## Data Availability

The datasets presented in this study were submitted to The National Center for Biotechnology Information (NCBI) database with the accession numbers OM421636 and OP154196.

## Supplementary Materials

The following supporting information can be downloaded at: https://www.mdpi.com/article/10.3390/ani13030362/s1, Figure S1: Inferred process of mitochondrial gene rearmament in both Ophichthus species; Figure S2: Amino acid compositions in the mitogenomes of two snake eels; Figure S3: Inferred secondary structures of the 22 tRNA genes of two *Ophichthus* species; Figure S4: The rates of non-synonymous substitutions (*Ka*) and synonymous substitutions (*Ks*) for each PCG in pairwise mitochondrial genome of *O. evermanni* and *O. erabo*; Table S1: Composition and skewness of mitogenomes in 25 Anguilliformes species with novel gene order; Table S2: The O_L_ structure in the mitogenomes of nine Ophichthidae fishes;
