# Supplementary material for: Comparative Mitogenomic Analysis of Two Snake Eels Reveals Irregular Gene Rearrangement and Phylogenetic Implications of Ophichthidae
Source: Animals (Basel). 2023 Jan 20;13(3):362. doi: 10.3390/ani13030362 (PMC9913227; doi:10.3390/ani13030362)
Supplement: Supplementary file 1 [file animals-13-00362-s001.zip › animals-2107475-supplementary.pdf]

Table S1. Composition and skewness of mitogenomes in 25 Anguilliformes species with novel gene order.

Family	Species	GenBank accession No.	Length / bp	A %	T %	G %	C %	A+T %	AT-skew	GC-skew
Chlopsidae	*Thalassenchelys* sp.	AP010867	17,617	31.96	24.37	16.13	27.54	56.33	0.135	-0.261
Colocongridae	*Coloconger cadenati*	AP010863	17,755	31.43	25.14	16.02	27.41	56.57	0.111	-0.262
Congridae	*Ariosoma shiroanago*	AP010861	16,922	32.54	26.85	16.71	23.90	59.38	0.096	-0.177
	Conger japonicus	KR131863	17,778	32.04	27.33	16.99	23.64	59.37	0.079	-0.164
	Conger myriaster	AB038381	18,705	33.89	29.29	14.70	22.12	63.18	0.073	-0.202
	Heteroconger hassi	AP010859	17,768	31.65	24.34	16.81	27.17	55.99	0.131	-0.236
	Paraconger notialis	AP010860	17,729	32.48	23.06	15.72	28.74	55.54	0.170	-0.293
Derichthyidae	*Derichthys serpentinus*	AP010851	17,077	31.37	24.27	16.20	28.17	55.64	0.128	-0.270
	Nessorhamphus ingoifianus	AP010850	17,782	32.66	25.09	15.26	26.99	57.74	0.131	-0.278
Muraenesocidae	*Cynoponticus ferox*	AP010853	17,822	32.43	27.38	15.21	24.99	59.81	0.084	-0.243
	Muraenesox bagio	AP010852	18,247	32.32	28.17	16.29	23.23	60.49	0.069	-0.176
	Muraenesox cinereus	MT571331	17,673	32.08	27.57	16.56	23.79	59.65	0.076	-0.179
Nettastomatidae	*Facciolella oxyrhyncha*	AP010866	17,789	32.98	25.79	16.12	25.10	58.77	0.122	-0.218
	Hoplunnis punctata	AP010865	17,828	33.85	24.21	15.56	26.39	58.05	0.166	-0.258
	Nettastoma parviceps	AP010864	17,714	31.57	26.79	16.82	24.83	58.35	0.082	-0.192
Ophichthidae	*Brachysomophis crocodilinus*	KY081398	17,818	32.11	24.69	16.22	26.98	56.80	0.112	-0.223
	Myrichthys maculosus	AP010862	17,859	31.20	24.99	16.82	26.98	56.19	0.102	-0.235
	Ophichthus brevicaudatus	MZ334613	17,771	30.93	26.15	16.43	26.49	57.08	0.086	-0.237
	Ophichthus erabo	OP154196	17,856	32.07	25.44	16.25	26.24	57.50	0.115	-0.235
	Ophichthus evermanni	OM421636	17,759	31.27	26.32	16.19	26.22	57.59	0.086	-0.237
	Ophichthus rotundus	KY081397	17,785	30.59	24.94	17.01	27.46	55.52	0.115	-0.235
	Ophisurus macrorhynchos	AP002978	17,843	32.18	25.72	16.36	25.74	57.90	0.084	-0.234
	Pisodonophis boro	AP019349	15,400	31.05	25.03	16.54	27.38	56.08	0.111	-0.232
	Pisodonophis cancrivorus	AP019350	17,746	31.74	26.25	15.61	26.41	57.98	0.131	-0.249

Table S2. The O_L structure in the mitogenomes of nine Ophichthidae fishes

Species	Location (5′-3′)	Lengt h (bp)	Stem (5′-3′)	Loop (5′-3′)	Stem (5′-3′)
Brachysomophis	5327-5353	27	CCGCCTCT	CCGGGGGAGTA	AGAGGCGG

crocodilinus					
Myrichthys maculosus	5336-5366	31	TCCCGCCTCTT	AGGGGGAGG	GGGAGGCGGGA
Ophichthus brevicaudatus	5341-5372	32	TCCCCCCCCCGTG	GGGGGA	CAGGGGGGGGGGA
Ophichthus erabo	5351-5376	26	CCGCCTCT	TAGGGGGAGA	GGAGGCGG
Ophichthus evermanni	5335-5368	34	CCCCGCCCCCT	GGGGGGGAAGGG	AGGGGGCGGGG
Ophichthus rotundus	5339-5360	22	CTCCCCCG	CCTCTC	CGGGGGAG
Ophisurus macrorhynchos	5336-5361	26	CCGCCTCTTC	GGGGGA	GAGGAGGCGG
Pisodonophis boro	5342-5363	22	CTCCCCCG	CCTCTC	CGGGGGAG
Pisodonophis cancrivorus	5329-5349	21	CTCCCCCG	CCTCC	CGGGGGAG

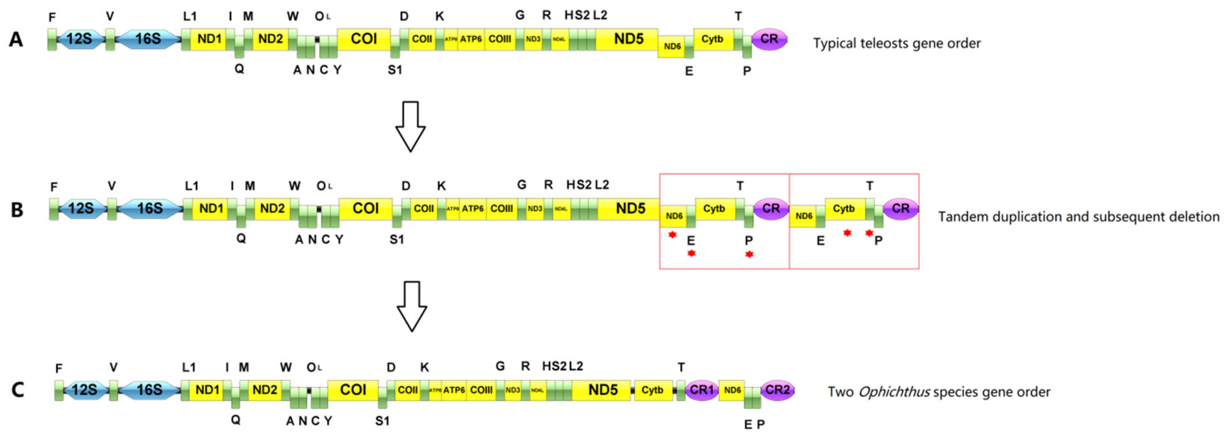

Figure S1. Inferred process of mitochondrial gene rearmament in both *Ophichthus* species. Gene cluster in red box was duplicated and the subsequent deletions of redundant genes were marked with star symbols.

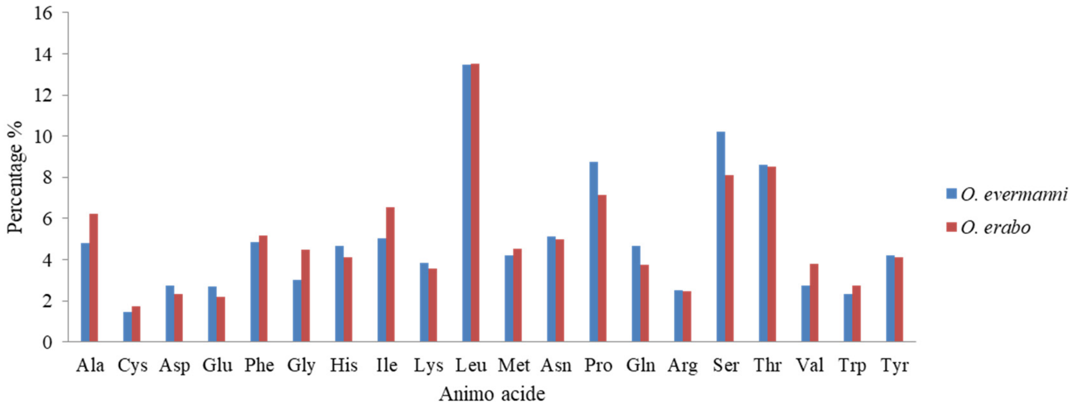

Figure S2. Amino acid compositions in the mitogenomes of two snake eels.

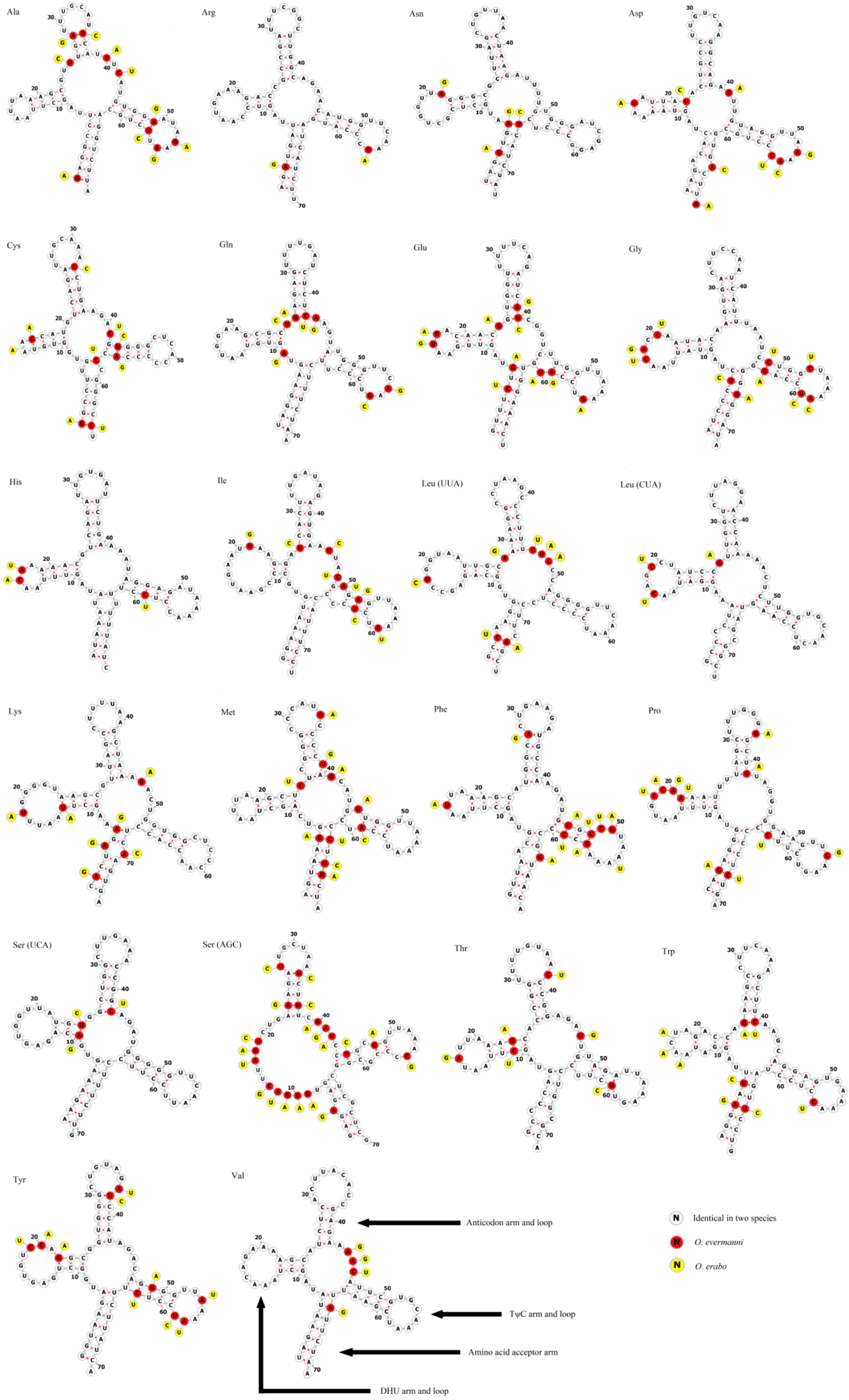

Figure S3. Inferred secondary structures of the 22 tRNA genes of two *Ophichthus* species.

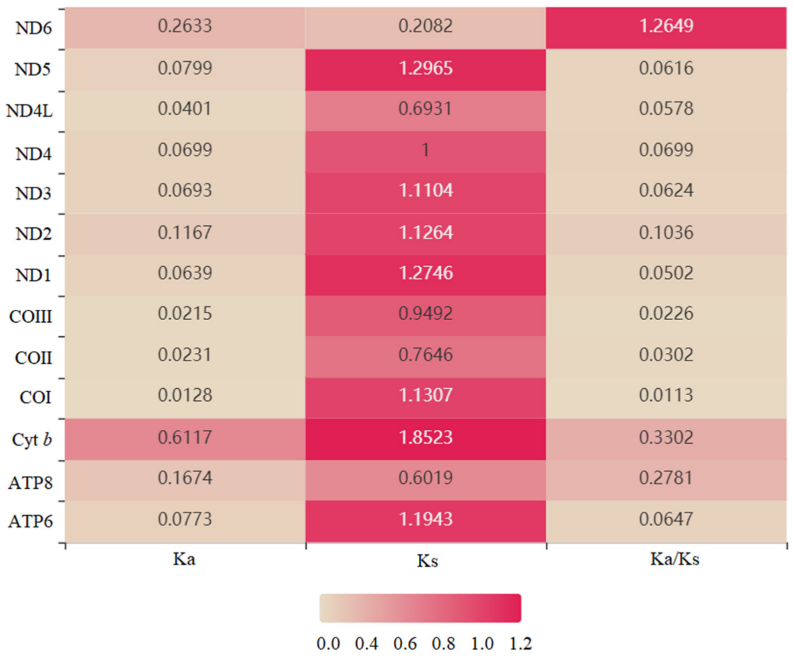

Figure S4. The rates of non-synonymous substitutions (*Ka*) and synonymous substitutions (*Ks*) for each PCG in pairwise mitochondrial genome of *O. evermanni* and *O. erabo*.
